# Virtual Screening against Phosphoglycerate Kinase 1 in Quest of Novel Apoptosis Inhibitors

**DOI:** 10.3390/molecules22061029

**Published:** 2017-06-21

**Authors:** Jie Xia, Bo Feng, Qianhang Shao, Yuhe Yuan, Xiang Simon Wang, Naihong Chen, Song Wu

**Affiliations:** 1 State Key Laboratory of Bioactive Substance and Function of Natural Medicines, Department of New Drug Research and Development, Institute of Materia Medica, Chinese Academy of Medical Sciences and Peking Union Medical College, Beijing 100050, China; jiexia@imm.ac.cn (J.X.); fengbo@imm.ac.cn (B.F.); 2 Department of Pharmacology, Institute of Materia Medica, Chinese Academy of Medical Sciences and Peking Union Medical College, Beijing 100050, China; shaoqianhang@imm.ac.cn (Q.S.); yuanyuhe@imm.ac.cn (Y.Y.); chennh@imm.ac.cn (N.C.); 3 Molecular Modeling and Drug Discovery Core Laboratory for District of Columbia Center for AIDS Research (DC CFAR), Department of Pharmaceutical Sciences, College of Pharmacy, Howard University, Washington, DC 20059, USA; x.simon.wang@gmail.com

**Keywords:** apoptosis, phosphoglycerate kinase 1, terazosin, virtual screening, Parkinson’s disease

## Abstract

Inhibition of apoptosis is a potential therapy to treat human diseases such as neurodegenerative disorders (e.g., Parkinson’s disease), stroke, and sepsis. Due to the lack of druggable targets, it remains a major challenge to discover apoptosis inhibitors. The recent repositioning of a marketed drug (i.e., terazosin) as an anti-apoptotic agent uncovered a novel target (i.e., human phosphoglycerate kinase 1 (hPgk1)). In this study, we developed a virtual screening (VS) pipeline based on the X-ray structure of Pgk1/terazosin complex and applied it to a screening campaign for potential anti-apoptotic agents. The hierarchical filters in the pipeline (i.e., similarity search, a pharmacophore model, a shape-based model, and molecular docking) rendered 13 potential hits from Specs chemical library. By using PC12 cells (exposed to rotenone) as a cell model for bioassay, we first identified that AK-918/42829299, AN-465/41520984, and AT-051/43421517 were able to protect PC12 cells from rotenone-induced cell death. Molecular docking suggested these hit compounds were likely to bind to hPgk1 in a similar mode to terazosin. In summary, we not only present a versatile VS pipeline for potential apoptosis inhibitors discovery, but also provide three novel-scaffold hit compounds that are worthy of further development and biological study.

## 1. Introduction

Apoptosis is the death of a cell regulated by a tight intracellular program (i.e., programmed cell death) [1]. The precise control of apoptosis is indispensable to maintain the homeostasis of development and the aging of tissues and normal immune defense, while the abnormal regulation of apoptosis could cause many human conditions [2]. Excessively activated apoptosis has been identified in the development of neurodegenerative disorders (e.g., Parkinson’s disease) [3], stroke [4], and sepsis [5]. Clearly, the inhibition of apoptosis is a potential therapeutic strategy to treat the above mentioned pathologies.

**Figure 1:**
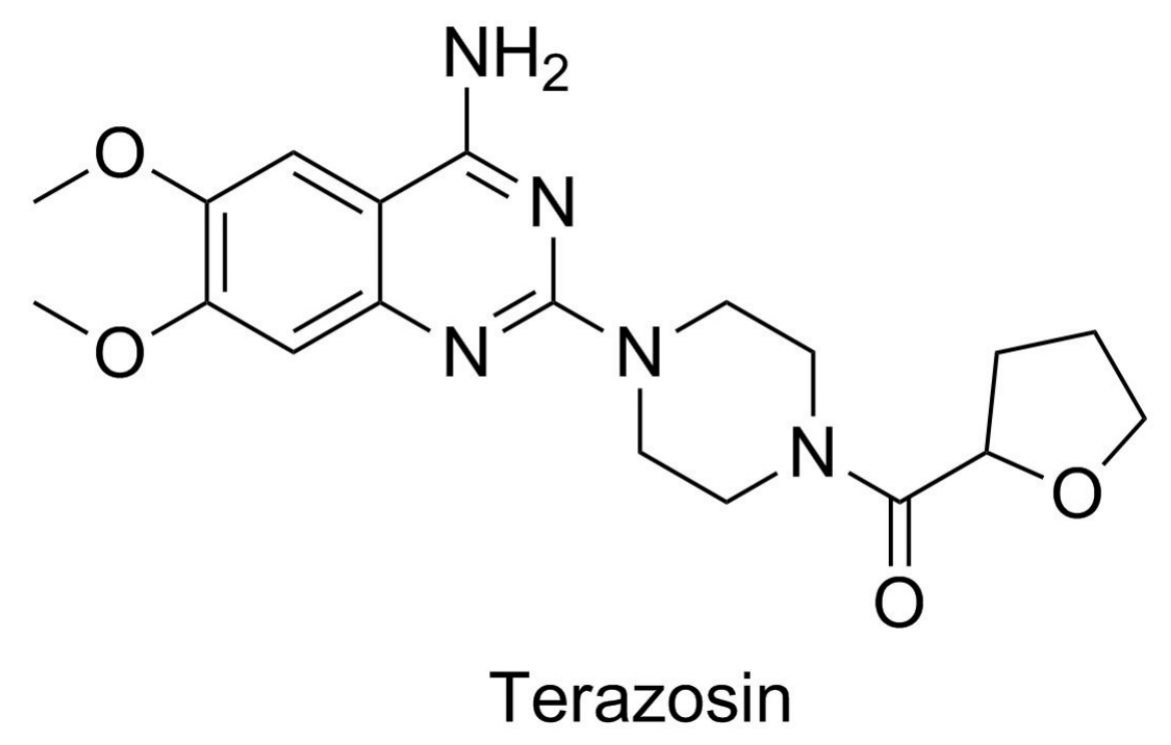
The chemical structure of terazosin that binds to hPgk1.

For a long time, the main targets for the discovery of small-molecule apoptosis inhibitors have been cysteine aspartate-specific proteases (caspases) that directly mediate apoptosis [6,7]. In the family of caspases, caspase-3 and caspases-7 were the most attractive ones [8,9,10,11]. Unfortunately, though great efforts were taken, no caspase inhibitor has been approved for clinical use so far [12]. Heat shock proteins (HSPs, e.g., HSP90, HSP70) constitute another potential class of targets. A few natural products (e.g., withanolides [13] and chalcones [14]) were reported to be HSP inducers that had potential cytoprotective effects [15,16,17,18]. Most recently, the Liu group repurposed a marketed drug (i.e., terazosin (cf. Figure 1)) as an anti-apoptotic agent in vitro and in vivo. They further discovered terazosin binds to phosphoglycerate kinase 1 (Pgk1) and is able to interact with HSP90, thus suggesting that Pgk1 may serve as a druggable target for apoptosis inhibitors discovery [12]. Evidence from other groups also confirmed the role of Pgk1 as a suppressor of apoptosis [19,20].

Pgk1 is a transferase and functions as an ATP-generating enzyme in glycolysis. Unlike its isozyme Pgk2 unique to spermatogenic cells, it is expressed in all cells in humans [21]. The previous study toward the precise catalytic mechanism of Pgk1 led to the release of multiple crystal structures [22,23,24]. However, those structures only uncovered the binding modes of ADP, ADP analogues and ATP. Encouragingly, the recent X-ray structure of the human Pgk1 (hPgk1)/terazosin complex (PDB Entry: 4O33) resolved by the Liu group addressed this dilemma, by providing much exploitable knowledge on interactions between hPgk1 and its activator [12].

Herein, we create FCFP_6 fingerprints from the two-dimensional structure of terazosin, generate a pharmacophore model based on the hPgk1-terazosin interactions, and build a shape-based model from the binding pose of terazosin. Then we construct a hierarchical VS pipeline by combining those ligand-based models and molecular docking. In order to validate the efficacy of the pipeline, we further apply it to screening the Specs chemical library and test potential hits for their preliminary protective effects on PC12 cells exposed to rotenone (a cell model of Parkinson’s disease).

## 2. Results and Discussions

### 2.1. FCFP_6 Fingerprint-Based Similarity Search

As structurally similar compounds exhibit similar biological activities [25,26], we set similarity search as the first step of the VS pipeline. This step aimed to identify compounds that shared common FCFP_6 fingerprints with terazosin from the Specs chemical library (~210,000), in a computationally inexpensive manner. At the end of this step, we kept the top 10,000 Specs compounds. Their FCFP_6 fingerprint-based similarity to terazosin (in terms of the Tanimoto coefficient) ranged from 0.444 to 0.145.

### 2.2. Pharmacophore Models and Database Filtering

**Figure 2:**
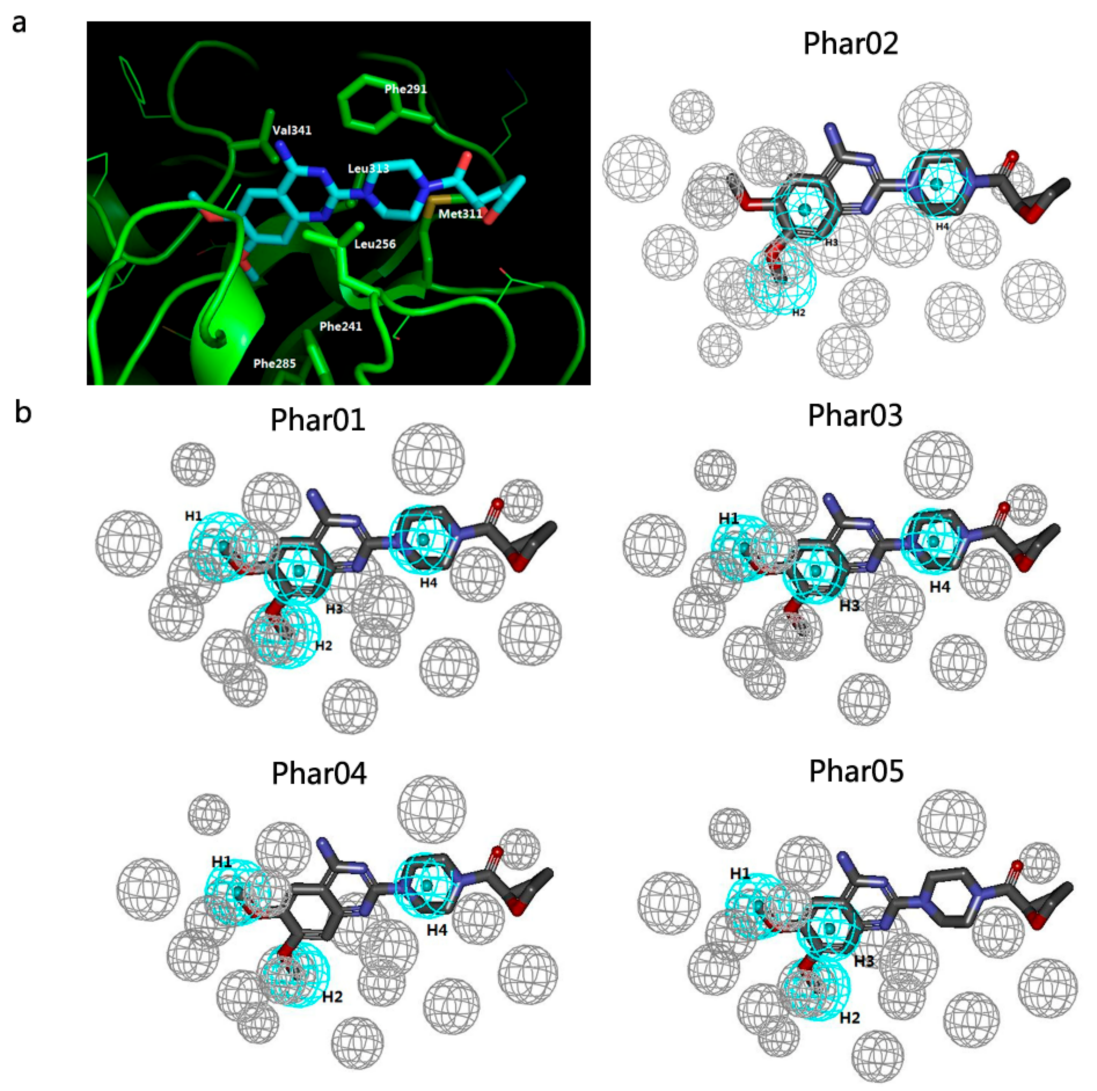
The pharmacophore models (based on protein-ligand interactions) generated by DS2016/Catalyst: (**a**) the binding mode of terazosin to hPgk1 (left panel), as well as the model selected for pharmacophore filtering (right panel); and (**b**) the other four pharmacophore models. Color codes: light blue, hydrophobic group; gray, excluded volumes.

DS2016/Catalyst identified 16 pharmacophore features in the chemical structure of terazosin, of which only four hydrophobic features matched the protein-ligand interactions between hPgk1 and terazosin. As each pharmacophore was allowed to contain a minimum of three and a maximum of six features, DS2016/Catalyst automatically generated five pharmacophore models. As shown in Figure 2, all the models (i.e., Phar01–Phar05) are composed of hydrophobic features and excluded volumes. To be specific, Phar01 consists of four hydrophobic features (i.e., H1H2H3H4) while others contain three hydrophobic features (i.e., Phar02/H2H3H4, Phar03/H1H3H4, Phar04/H1H2H4, and Phar05/H1H2H3). We then carefully inspected the binding mode of terazosin and the position of each hydrophobic feature and observed: (1) the piperazine ring of terazosin is located in a hydrophobic site surrounded by Leu256, Phe291, Met311, and Leu313. Thus, H4 from the piperazine ring was essential for ligand binding; (2) as demonstrated by Chen X. et al., the six-membered benzene ring of terazosin’s quinazoline core shares the site with the six-membered pyrimidine ring of the purine in ADP/ATP [12]. Due to the importance of this binding mode to the activation of hPgk1, H3 at that site was considered indispensible; (3) as the methoxy group corresponding to H2 instead of H1 stretched into the subpocket and formed clear hydrophobic interactions with Phe241 and Phe285, H2 seemed to be more important than H1. Based on these observations, we selected Phar02 as the model for pharmacophore filtering.

A total of 14,932 chemical entities (i.e., protonated states and isomers of 10,000 unique compounds from similarity search) were submitted for pharmacophore filtering through Phar02. From all the matched ones, 10,000 chemical entities (including 7278 unique compounds) were selected. Their FitValue scores ranged from 3.000 to 1.969.

### 2.3. Shape-Based Model and Database Filtering

**Figure 3:**
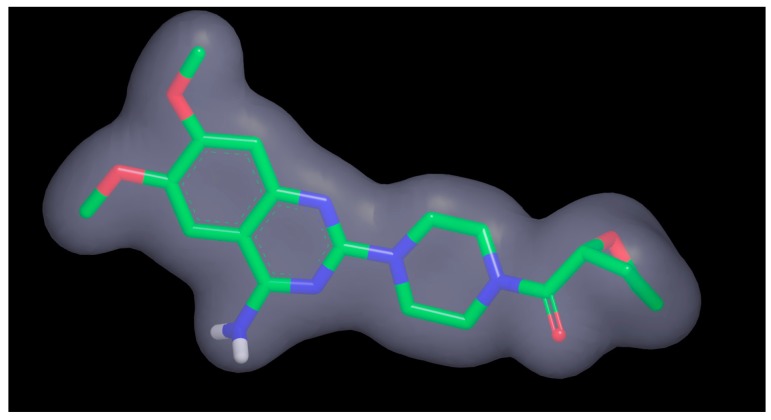
The shape-based model generated from terazosin by ROCS program. Color code: gray, shape-based model.

The shape-based model was based on the native pose of terazosin bound to hPgk1 (cf. Figure 3). Though the pharmacophore features were also identified from the chemical structure of terazosin, they were not considered in the shape-based filtering. As described in the Method section, the top 5000 chemical entities were retained and they matched the shape-based model in ShapeTanimoto values from 0.899 to 0.685. The chemical entities covered 3956 Specs unique compounds.

### 2.4. Potential Hits from Molecular Docking

**Figure 4:**
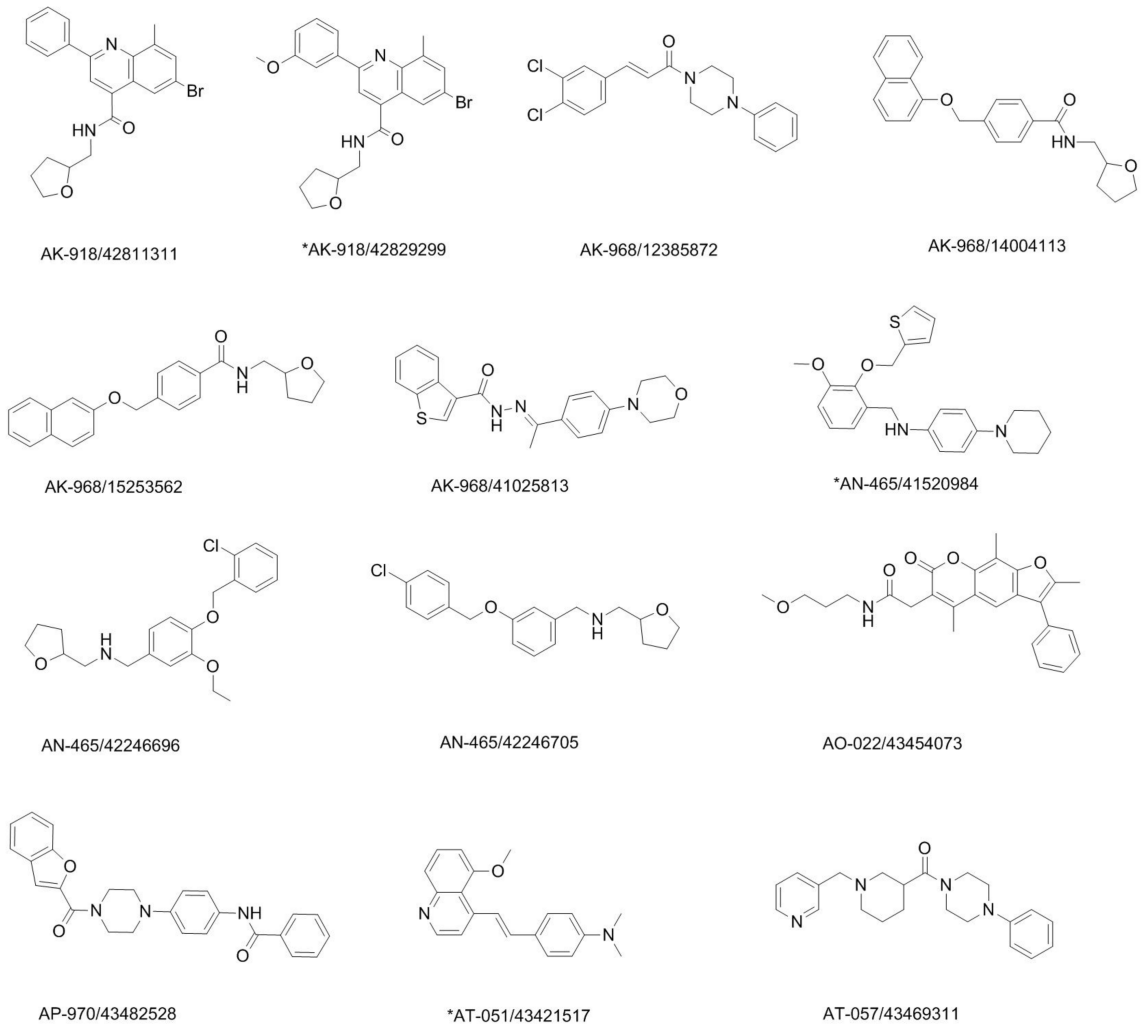
The chemical structures of 13 potential hits (note: the compounds labeled with “*” were experimentally validated as active).

**Figure 5:**
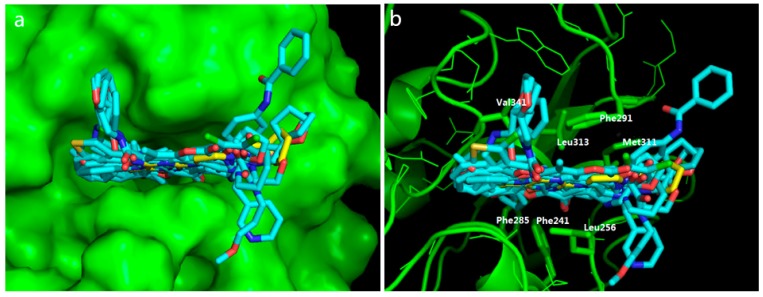
The binding poses of 13 potential hits: (**a**) the binding pocket of hPgk1 by the surface representation; and, (**b**) protein-ligand interactions between the potential hits and hPgk1. Color codes: green, hPgk1; light blue, potential hits; yellow, terazosin; red, oxygen atom; dark blue, nitrogen atom.

After “fast docking” and “precise docking” against the protein structure of hPgk1 by GOLD, we retained 500 top-ranking compounds (~12.6%) from the docked 3956 unique ones. Based on their predicted binding affinity in terms of ChemScore and binding modes, we further selected 13 compounds as potential hits for experimental validation. Their chemical structures, ChemScore values, and binding poses are shown in Figure 4, Table 1, and Figure 5, respectively. The ChemScore values greater than 32.052 (as well as the ChemScore ranks in the top 221) of these potential hits are indicative of relatively strong binding affinity to hPgk1. Besides, all compounds occupied the same binding site as terazosin (cf. Figure 5a) and formed hydrophobic interactions with Val341, Phe285, Phe241, Leu256, Met311, Leu313, or Phe291 (cf. Figure 5b). The potential hits were from the FCFP_6 fingerprint-based similarity search and pharmacophore filtering and shape filtering; thus, they undoubtedly shared a few common features with terazosin (e.g., fingerprints, and hydrophobic group). However, their 2D similarity value of less than 0.205 was indicative of structural novelty (cf. Table 1).

### 2.5. Protective Effect against Rotenone-Induced PC12 Cell Death

**Figure 6:**
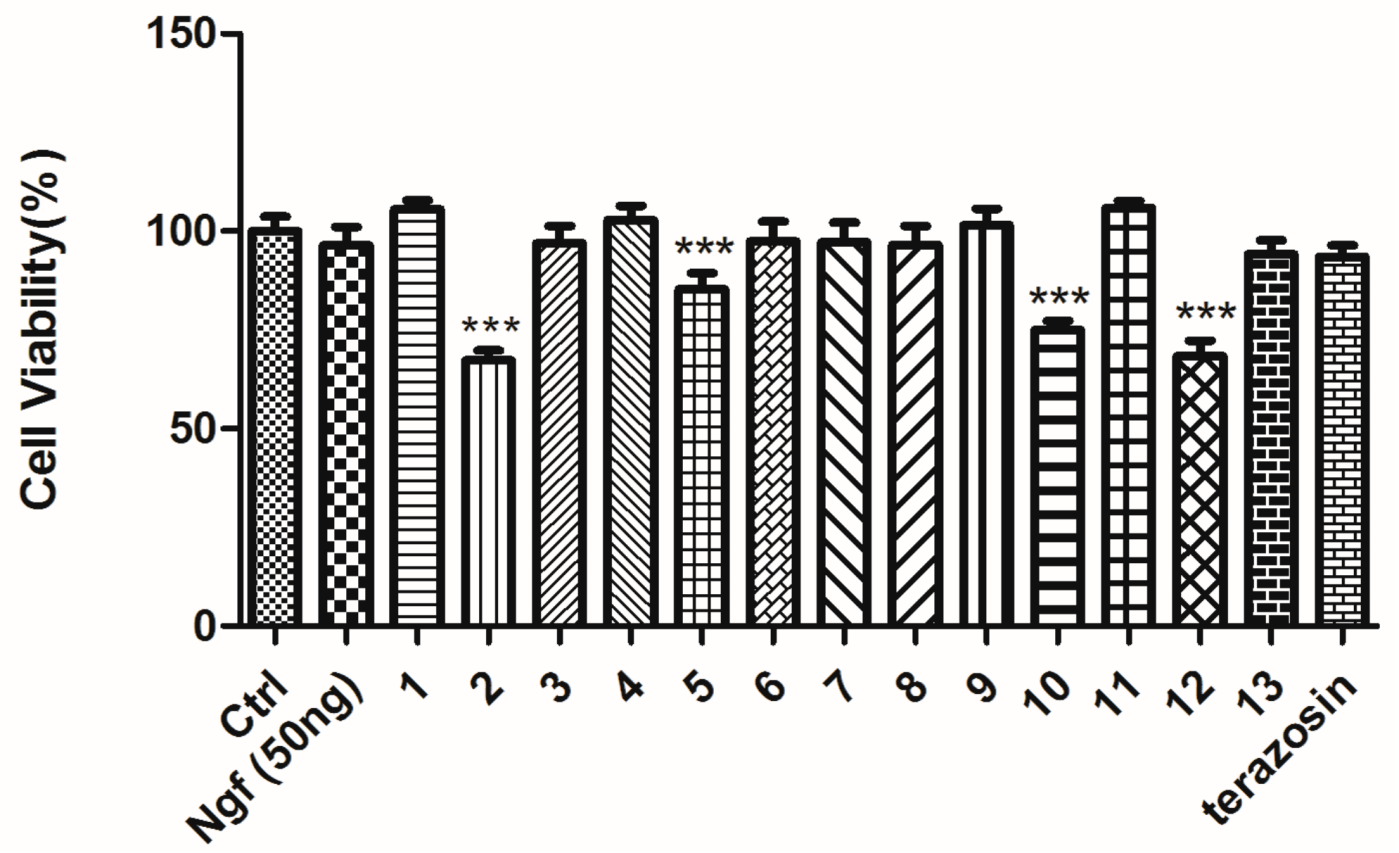
The effects of 13 potential hits on PC12 cells in the absence of rotenone. Ctrl, PC12 cells incubated with 0.1% DMSO only; Ngf, treatment of 50 ng/mL nerve growth factor; terazosin, treatment of 10 μmol/L terazosin. Error bars represent SD. *** *p* <0.001 vs. control group; one-way analysis of variance was used (*n* = 9).

As an experimental validation, we tested the protective effects of 13 potential hits on PC12 cells exposed to rotenone. As a neurotoxin and mitochondrial complex I inhibitor, rotenone induces apoptotic cell death by enhancing the formation of mitochondrial reactive oxygen species (ROS) [27,28]. Thus, rotenone is widely used to establish in vitro and in vivo models for Parkinson’s disease. The PC12 cell model exposed to rotenone was one of them, with which we have studied pharmacology for multiple compounds [29,30]. In the present work, we used 4 μmol/L of rotenone to incubate with the PC12 cells and established the disease model. As shown in Table 2, rotenone reduced a survival rate of PC12 cells significantly, from 100.0 ± 4.203% (control) to 73.84 ± 4.632% (model). After the treatment of a positive drug (i.e., nerve growth factor (NGF) (50 ng/ml) [31]), the survival rate was restored. These data indicated the reliability of our rotenone-induced PC12 cell death model. By comparing the cell viability of PC12 cells treated with rotenone, with rotenone plus compounds, we found that AN-465/41520984, AK-918/42829299, and AT-051/43421517 potentially protected PC12 cells against rotenone-induced cell death by increasing cell viability. Furthermore, none of them showed statistically significant improvement of PC12 cell viability in the absence of rotenone (cf. Figure 6). It excluded the likelihood of them inducing cell proliferation (that may also enhance cell viability). Therefore, these preliminary data clearly demonstrated the protective effects of three Specs compounds (i.e., AN-465/41520984, AK-918/42829299, and AT-051/43421517). Then, we retrieved the PubChem database for bioactivity data associated with these hit compounds [32,33,34]. Encouragingly, none of them has been reported before as either protective agents or apoptosis inhibitors. We also tested the effect of terazosin on the rotenone-induced PC12 cell injury model. Unfortunately, terazosin was not able to attenuate the effect of rotenone by enhancing cell viability at the same concentration (i.e., 10 μM).

**Figure 7:**
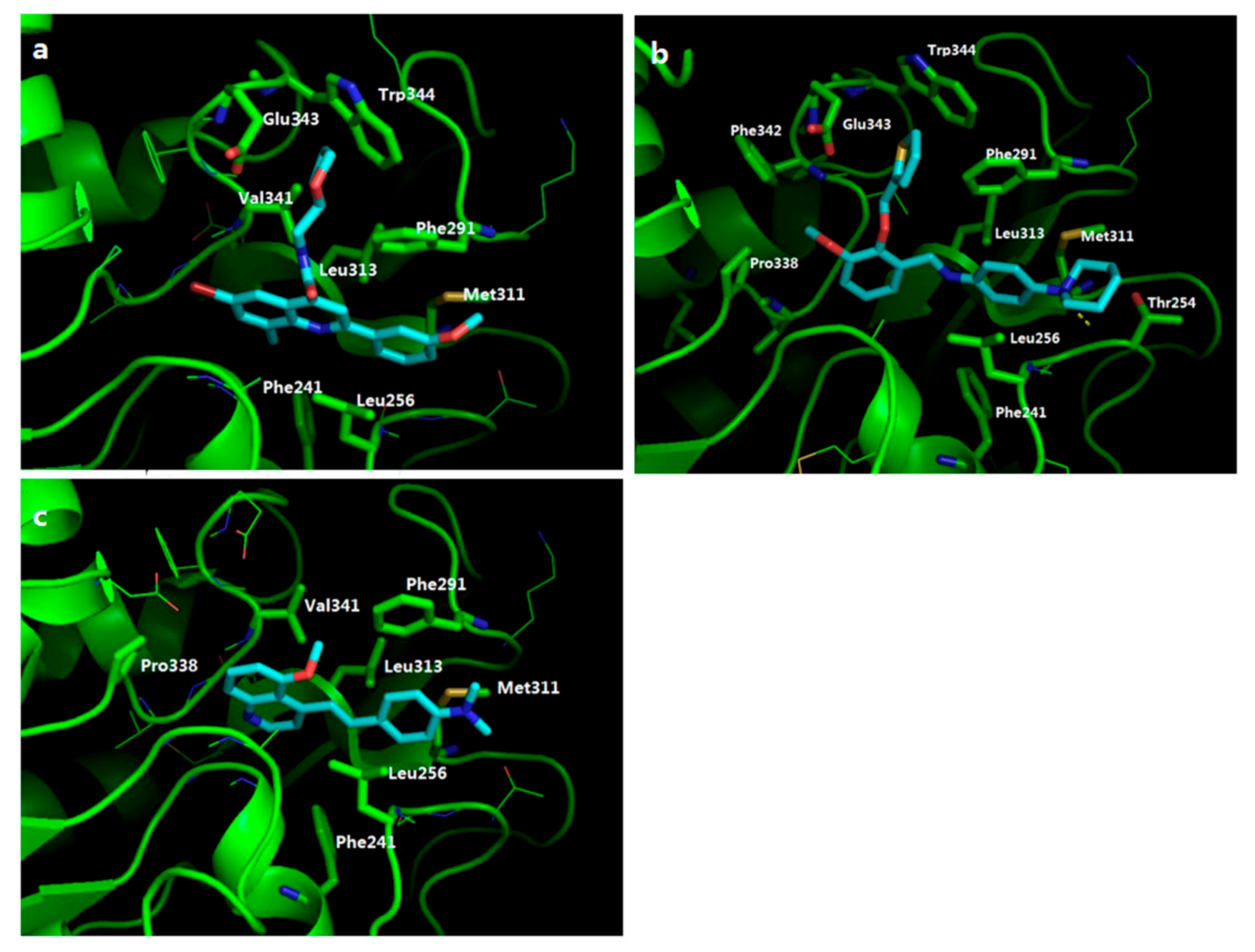
The predicted binding modes of three apoptosis inhibitors to hPgk1: (**a**) AK-918/42829299; (**b**) AN-465/41520984; and, (**c**) AT-051/43421517. The residues that interact with each hit compound are labeled. Color codes: green, hPgk1; light blue, apoptosis inhibitors; red, oxygen atom; dark blue, nitrogen atom; yellow, sulfur atom.

Though there is currently no available data that indicated that the three hit compounds truly targeted hPgk1, the molecular docking showed they bound to hPgk1 in a favorable way (cf. Figure 7). Firstly, all these hit compounds occupied the binding site of terazosin by forming π-π stacking with Phe291 and hydrophobic interactions with Leu256, Met311, and Leu313. Secondly, every compound contained a substituted group that extended into the small pocket surrounded by Val341, Trp 344, and Phe 291 (e.g., (tetrahydrofuran-2-yl)methyl group of AK-918/42829299, (thiophen-2-yl)methyl group of AN-465/41520984, or methyl group of AT-051/43421517). These substituted groups formed hydrophobic interactions with hPgk1, and can thus enhance the binding of the hit compounds.

## 3. Materials and Methods

### 3.1. The General Workflow for Drug Discovery

**Scheme 1:**
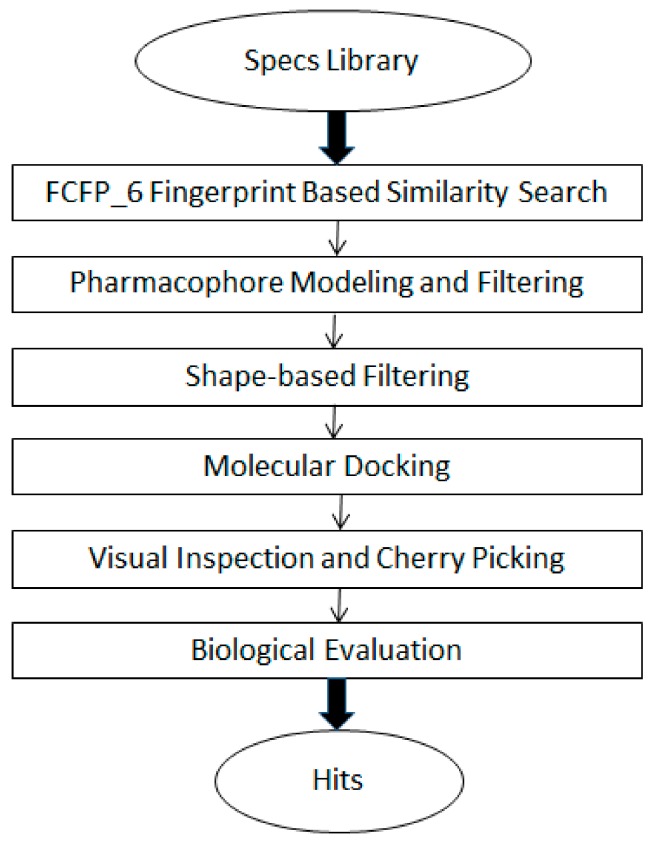
The general workflow for the discovery of apoptosis inhibitors.

The workflow for discovery of potential apoptosis inhibitors included a VS pipeline and preliminary biological evaluation (cf. Scheme 1). The VS pipeline was composed of five consecutive steps: (1) the FCFP_6 fingerprint-based similarity search using the two-dimension structure of terazosin as a reference; (2) filtering by a pharmacophore model, constructed based on the interactions between terazosin and hPgk1; (3) filtering by a shape-based model from the native pose of terazosin to hPgk1, i.e., the pose in the X-ray structure; (4) molecular docking against the protein structure of hPgk1; and, (5) visual inspection and cherry picking of potential hits. The input of this workflow was the Specs chemical library (http://www.specs.net/, accessed by November 2015), which contained approximately 210,000 compounds. The outputs from the VS workflow were potential hPgk1 binders/apoptosis inhibitors. A cell model of Parkinson’s disease (i.e., PC12 cells exposed to rotenone) was used for preliminary bioassay. To be specific, the protective effects of those compounds from rotenone-induced neurotoxicity were tested.

### 3.2. FCFP_6 Fingerprint-Based Similarity Search

The “Find Similar Molecules by Fingerprints” module in Discovery Studio 2016 (DS2016, Dassault Systèmes BIOVIA, San Diego, CA, USA) was applied for the similarity search. The chemical structure of terazosin was set as the query/reference ligand. The Specs compound library (composed of approximately 210,000 molecules) was used as a screening dataset. The two-dimension structures of both the query ligand and the Specs compounds were coded by FCFP_6 fingerprints. The similarity of each Specs compound to the query ligand was measured by the Tanimoto coefficient. According to the similarity score, top 10,000 Specs-unique compounds were retained for further analysis.

### 3.3. Pharmacophore Modeling and Filtering

#### 3.3.1. Receptor-Ligand Pharmacophore Generation

The X-ray structure was retrieved and downloaded from the Protein Data Bank (PDB). The co-crystallized water molecules and 3-phosphoglyceric acid were removed from the X-ray structure, while the structure of the cognate ligand (i.e., terazosin) was kept. The structure of human hPgk1 was then prepared by using the “Clean Protein” module of DS2016. This module automatically added hydrogen atoms, modified chain termini, corrected nonstandard names, repaired incomplete residues, and atom order in amino acids, and also protonated the whole protein at pH 7.0. Based on the prepared hPgk1/terazosin complex, the module named “Receptor-Ligand Pharmacophore Generation” in DS 2016/Catalyst was used to generate structure-based pharmacophore models [35]. Herein, the maximum number of pharmacophore models generated by the module was set at 10. And the minimum and maximum numbers of pharmacophore features in each model were set to 3 and 6, respectively. Shape constraint was not added to the pharmacophore. The selectivity of each model was scored by a scoring function, based on a genetic function approximation (GFA) model. After pharmacophore modeling, the best model was selected based on the selectivity score, as well as the interactions between hPgk1 and terazosin.

#### 3.3.2. Construction of a Multi-Conformation Ligand Database

The outputs from the FCFP_6 Fingerprint-based similarity search (i.e., 10,000 Specs compounds) were prepared by using the “Prepare Ligands” module in DS 2016. With this module, the protonated states of each compound at the pH range of 7.3–7.5 were generated, and all potential isomers were also enumerated. Besides, the lowest-energy conformation was generated for each chemical entity (i.e., protonated state or isomer) by a built-in Catalyst program (note: the Lipinski filter was not applied here). Then, the DS2016 module “Build 3D Database” was used to convert the pool of chemical entities to a multi-conformation ligand database. For that purpose, the “FAST” algorithm was selected and a maximum of 100 conformations was generated for each chemical entity.

#### 3.3.3. Pharmacophore Filtering

The “Search 3D Database” module in DS2016 was run to perform pharmacophore filtering. To be specific, a rigid fit of the ligand conformations was performed against the best pharmacophore model. The score (named FitValue) measured how well the ligand fit the pharmacophore. Top 10,000 chemical entities of high scores were retained after pharmacophore filtering.

### 3.4. Shape-Based Filtering

Firstly, ROCS (version 3.2.1, OpenEye Scientific Software Inc., Santa Fe, NM, USA) [36,37] was applied to build the shape-based filter. This program automatically recognized and generated the shape and pharmacophore features from the native pose of terazosin. Secondly, a multi-conformer database of the 10,000 chemical entities was built by OMEGA (version 2.5.1.4, OpenEye Scientific Software, Inc.) [38]. The default number of conformations for each chemical entity was up to 200. Lastly, the multi-conformer database was screened using the shape as the query. During the screening, a shape-only overlay as well as a full-best overlay optimization was performed. The score to measure shape similarity was the ShapeTanimoto coefficient. 5000 chemical entities were kept from shape-based filtering and their corresponding Specs IDs were retrieved.

### 3.5. Molecular Docking

All the chemical entities corresponding to those unique IDs were docked against the prepared protein structure by using GOLD (version 3.0.1, Cambridge Crystallographic Data Center, Cambridge, UK) [39]. Firstly, “7–8 times speedup” settings for Genetic Algorithm (GA) Parameters were used so as to run a “fast docking”. The binding site was defined as a sphere centered on terazosin with a radius of 10 Å; for every ligand, the times for molecular docking were 10. However, molecular docking was allowed to stop if the first three docking poses were within 1.5 Å RMSD. In each time, the binding affinity of the chemical entity to the target protein was predicted by ChemScore. For each chemical entity, all docking poses were ranked by ChemScore and its highest-scoring pose was retained. The highest score represented the predicted binding affinity of the chemical entity. In the situation where multiple chemical entities corresponded to one Specs compound/ID, only the highest-scoring one was kept for that Specs compound. In this way, each Specs compound/ID corresponded to one ChemScore. Then, all the Specs compounds were ranked according to the ChemScore. A total of top 500 Specs compounds were kept for a “precise docking” (herein, “precise docking” means the use of default settings for GA Parameters, which delivered high-predictive accuracy but were relatively slow). The parameters (except for the ones above) were the same as those used for the “fast docking”. Prior to “precise docking”, the Specs compounds were also prepared. After docking, only the highest-scoring pose/chemical entity was retained for each Specs compound.

### 3.6. Visual Inspection and Cherry-Picking of Potential Hits

The ChemScore values of 500 Specs compounds were ranked. The highest-scoring pose of each Specs compound was visually inspected. The criteria were: (1) the same binding site as terazosin; and, (2) hydrophobic interactions with Val341, Phe285, Phe241, Leu256, Met311, Leu313, or Phe291. The compounds with relatively high ranks (as well as a similar binding mode as terazosin) were picked for bioassay.

### 3.7. Biological Evaluation

#### 3.7.1. Reagents

Rotenone, dimethyl sulfoxide (DMSO), and 3-(4,5-Dimethylthiazol-2-yl)-2,5-diphenyl-tetrazolium bromide (MTT) were purchased from Sigma Chemical Company (St. Louis, MO, USA). 13 potential hits were purchased from Compound Handling B.V. (Zoetermeer, South Holland, The Netherlands).

#### 3.7.2. Cell Culture and Drug Treatment

PC12 cells were provided by the American Type Culture Collection (ATCC, Manassas, VA, USA). The cells were cultured in Dulbecco’s Modified Eagle’s Medium (DMEM) (Invitrogen Carlsbad, CA, USA), containing 5% fetal bovine serum, 5% heat-inactivated equine serum, and 100 U/mL of penicillin (as well as 100 μg/mL streptomycin) and cultured in a humidified incubator with 5% CO_2_ and 95% air at 37 °C. Cells were plated on 96-well plates at a density of 5 × 10^4^ cells/mL for 24 h. Rotenone and compounds (including NGF) were firstly dissolved in DMSO and then diluted to the specified concentration. In the primary bioassay, PC12 cells incubated with 0.1% DMSO were used as a control, while cells treated with 4 μmol/L rotenone were used as the disease model. PC12 cells (treated with both 4 μmol/L rotenone and 10 μmol/L compounds for 48 h) were used to explore potential effects of compounds on rotenone-induced PC12 cell death. In a secondary bioassay, PC12 cells incubated with 0.1% DMSO remained to be the control group, while the disease model (i.e., the rotenone-treated PC12 cell model) was not used. PC12 cells were treated with 10 μmol/L compounds alone. This assay was used to test the putative effects of compounds on cell proliferation.

#### 3.7.3. Cell Viability Assay

Cell viability was analyzed by the MTT assay. After incubation with 10 μL of 5 mg/mL MTT solution for 4 h at 37 °C, the formazan crystals were solubilized in 100 μL of MTT-formazan dissolving solution for 8–12 h. The optical density (OD) was measured at 570 nm using a Microplate Reader (Thermo, Waltham, MA, USA). Cell viability was expressed as a percentage (compared with the control).

## 4. Conclusions

In this study, we have constructed a hierarchical VS pipeline (specific to hPgk1) by making the best of a recently resolved X-ray structure of hPgk1-terazosin complex. The constructed pipeline consisted of three ligand-based approaches (i.e., similarity search, pharmacophore filtering, and shape-based filtering), along with a structure-based approach (i.e., molecular docking). We have used that pipeline to screen the Specs chemical library, cherry-picked 13 molecules for purchase, and tested their preliminary protective effects on PC12 cells exposed to rotenone (a cell model of Parkinson’s disease). The bioassay has shown three Specs compounds (i.e., AK-918/42829299, AN-465/41520984, and AT-051/43421517) are able to protect PC12 cells from rotenone-induced toxicity, thus validating the efficacy of our constructed pipeline. Based on the fast and cost-effective feature of our constructed pipeline, we anticipate its wide application to real-world screening.

To the best of our knowledge, the protective effect of the hit compounds has never been reported, and their chemical structures are diverse compared to terazosin. These features have demonstrated their potential to be starting structures for the development of potential apoptosis inhibitors. Though their true mechanism of action remains elusive, molecular docking has shown that the identified hit compounds tightly bind to hPgk1 in a mode similar to terazosin, indicating their likelihood of being hPgk1 activators. The immediate work will be the elucidation of their mechanism of action (e.g., whether the protective effect truly stems from the activation of hPgk1 as well as apoptosis inhibition).

## Figures and Tables

**Table 1:** Scores and ranks of 13 potential hits, in terms of Chemscore (GOLD), 2D similarity (FCFP_6), FitValue (Catalyst), and ShapeTanimoto (ROCS).

SPECS ID	Chemscore (GOLD)		2D Similarity (FCFP_6)		FitValue (Catalyst)		ShapeTanimoto (ROCS)	
	Score	Rank	Score	Rank	Score	Rank	Score	Rank
AK-918/42811311	34.820	50	0.178	1640	1.974	7269	0.704	3407
AK-918/42829299	35.759	25	0.205	312	2.869	2683	0.687	3899
AK-968/12385872	34.693	53	0.149	7964	2.530	5683	0.804	368
AK-968/14004113	35.193	31	0.180	1429	2.564	5495	0.770	1232
AK-968/15253562	35.815	23	0.192	707	2.978	470	0.743	2095
AK-968/41025813	34.493	57	0.150	7767	2.323	6411	0.758	1615
AN-465/41520984	37.517	2	0.147	9044	2.727	4366	0.726	2666
AN-465/42246696	34.955	40	0.162	3868	2.255	6597	0.770	1229
AN-465/42246705	33.160	143	0.158	4696	2.853	2946	0.749	1919
AO-022/43454073	37.586	1	0.155	5656	2.841	3091	0.787	719
AP-970/43482528	35.452	26	0.157	4923	2.754	4125	0.714	3071
AT-051/43421517	33.379	122	0.149	8073	2.997	52	0.748	1946
AT-057/43469311	32.052	221	0.162	3838	2.205	6741	0.755	1711

**Table 2:** The effect of 13 potential hits on rotenone-induced PC12 cell death.

Compound No.	Specs ID	Cell Viability (% of Control)
	control ^a^	100.0 ± 4.203
	model ^b^	73.84 ± 4.632 ###
	NGF ^c^	114.5 ± 3.704 ***
**1**	AN-465/41520984	89.66 ± 5.859 ***
**2**	AO-022/43454073	53.01 ± 3.772 ***
**3**	AK-968/15253562	78.43 ± 5.533
**4**	AK-918/42829299	88.04 ± 4.888 ***
**5**	AP-970/43482528	68.09 ± 4.812
**6**	AK-968/14004113	74.21 ± 3.454
**7**	AK-918/42811311	72.97 ± 3.662
**8**	AK-968/12385872	78.97 ± 7.843
**9**	AN-465/42246696	74.33 ± 10.64
**10**	AK-968/41025813	62.47 ± 7.143 ***
**11**	AT-051/43421517	87.97 ± 7.818 ***
**12**	AN-465/42246705	80.22 ± 6.887
**13**	AT-057/43469311	78.37 ± 6.860
	terazosin ^d^	72.25 ± 5.490

^a^ PC12 cells incubated with 0.1% DMSO only; ^b^ PC12 cells treated with 4 μmol/L rotenone; ^c^ PC12 cells treated with 4 μmol/L rotenone plus 50 ng/mL nerve growth factor (a positive drug that is able to improve cell viability); ^d^ for comparison purpose only; ### *p* < 0.001 vs. control, *** *p* < 0.001 vs. model group; one-way analysis of variance was used (*n* = 9).

## Abbreviations

Pgk1	phosphoglycerate kinase 1
VS	virtual screening
caspases	cysteine aspartate-specific proteases
HSPs	heat shock proteins
hPgk1	human Pgk1
ROS	reactive oxygen species
NGF	nerve growth factor
DS2016	discovery studio 2016
GFA	genetic function approximation
GA	genetic algorithm
DMSO	dimethyl sulfoxide
MTT	3-(4,5-Dimethylthiazol-2-yl)-2,5-diphenyl-tetrazolium bromide
ATCC	American type culture collection
DMEM	Dulbecco’s modified Eagle’s medium
OD	optical density
